# Adolescents’ Attachment to Parents and Reactive–Proactive Aggression: The Mediating Role of Alexithymia

**DOI:** 10.3390/ijerph182413363

**Published:** 2021-12-18

**Authors:** Elisa Mancinelli, Jian-Bin Li, Adriana Lis, Silvia Salcuni

**Affiliations:** 1 Department of Developmental Psychology and Socialization, University of Padua, 35131 Padua, Italy; adriana.lis@unipd.it (A.L.); silvia.salcuni@unipd.it (S.S.); 2 Digital Health Lab, Centre for Digital Health and Wellbeing, Fondazione Bruno Kessler, 38123 Trento, Italy; 3 Department of Early Childhood Education, The Education University of Hong Kong, Hong Kong, China; lijianbin@eduhk.hk

**Keywords:** reactive aggression, proactive aggression, alexithymia, attachment, adolescence

## Abstract

Aggressive behaviors can serve different functions, which might be understood by distinguishing between reactive (RA) and proactive (PA) aggression. Few studies were conducted on adolescents’ family precursors and emotional processes associated with RA or PA. Accordingly, the current study compared RA and PA by evaluating their association with adolescents’ attachment to parents and alexithymia. *N* = 453 Italian adolescents aged 15–19 years (Mage = 16.48; SD = 0.69; 33.6% males) participated in the study filling in self-report measures. Results showed that PA and RA are significantly associated and that PA was higher among males. Moreover, four mediational models were performed to assess the influence of adolescents’ attachment to mothers vs. fathers on RA or PA, considering the mediating role of alexithymia. Gender was included as a covariate. Mediational models’ results showed a direct and indirect effect, through lower alexithymia, of adolescents’ attachment to mothers and fathers on RA. Differently, only attachment to mothers showed a direct effect on PA, while attachment to fathers only an indirect effect, mediated by lower alexithymia, on PA was shown. Findings support the greater relevance of emotional processes for RA while highlighting the differential contribution of adolescents’ attachment to mothers vs. fathers upon PA. Implications are discussed, and suggestions for future research are provided.

## 1. Introduction

### 1.1. Adolescence, Reactive Aggression and Proactive Aggression

Adolescence represents the transition period between childhood and adulthood characterized by multiple changes at the biopsychosocial level, which further foresees a detachment from the parental figures to allow adolescents’ separation and individuation [1]. The unbalanced maturation between the higher-order cerebral structures responsible for emotional and behavioral regulation occurs simultaneously, and the sub-cortical, emotion-driven structures influence both emotional and behavioral responses [2,3]. Because of this unbalance in cerebral maturation, adolescents still present poor regulation capacities and thus heightened emotional responses resulting from an incapacity to properly mentalize their emotions and related physiological activation [3]. This predisposes them towards poor decision-making and renders them more reactive toward increased arousal as well as at an overall greater risk for risk-taking behaviors and adjustment problems [3]. Indeed, poor regulation capacities limit adolescents’ capacity to modulate their emotional reactivity and responsiveness, which is important for their anger expression and predicting their aggressiveness levels, and is in turn associated with social deviance and antisocial behaviors later in adulthood [4].

Aggression refers to behaviors aimed at harming people and/or objects that can be manifested directly or indirectly through physical or verbal acts [5]. Aggressive behaviors can serve different functions, which can be summed up by differentiating between reactive (RA) and proactive (PA) aggressive behaviors [5,6,7]. Specifically, RA can be defined as “impulsive aggression” as it usually occurs as an impulsive reaction to a perceived threat or provocation, indeed associated with high emotional and physiological arousal [6,7,8]. The conceptualization of RA is thus coherent with the frustration–aggression model, which suggests that aggression represents a reaction to frustrations and is directed either toward the frustration, eliciting situation, or to others, although not directly involved [7,9]. PA, on the other hand, regards “instrumental aggression” and thus describes the deliberate and purposeful display of aggressive behaviors aimed at reaching a goal, as being planned and generally motivated by the anticipation of a reward [6,10,11]. Differently from RA, PA can thereby be better understood through the social learning theory. This theory sees aggressive behaviors learned through modeling and vicarious reinforcement, whereby the aggressive behavior observed is seen in association with the obtention of a desired goal or objective [12,13].

Empirically, in adolescence, RA and PA were shown to be highly correlated [5,6,14,15], with correlations ranging between r = 0.60 and r = 0.80 [16,17]; however, their distinction is still debated [7,14,18,19]. Accordingly, although RA and PA present shared correlates such as increased drug use and parental violence [20], other research studies have instead stressed the importance of distinguishing between them since they present different correlates and are shown leading to different outcomes [6,7,21]. Indeed, albeit PA and RA present shared correlates [20], research evidence has also highlighted relevant differences. Notably, PA, compared to RA, is greatly linked with reduced cognitive and affective empathy [10,22,23], measures of psychopathy [23,24], and with serious delinquency referred to initiating fights, serious violent offending [6], and risky sexual behavior [16]. Differently, RA was shown to be greatly associated with impulsivity [6,25], hyperactivity, low IQ [20,22], negative emotionality [26], and poor emotion regulation [3,23].

Further differences between RA and PA that emerged in the literature regard gender differences. Specifically, it is noteworthy that males were altogether deemed as more aggressive than females both in adolescence and adulthood [20,27,28]; however, specific gender differences in RA and PA during adolescence are still contradicting [15,17,20,29,30]. For instance, Connor and colleagues [20] did not observe any gender difference in adolescents’ rates and severity of either RA or PA. Differently, Kempes, de Vries, and van Engeland [29], although similarly observing no gender differences in PA, instead reported greater RA among males compared to females. Others found that both RA and PA were significantly higher among male adolescents than the female counterparts [15,30].

### 1.2. The Association between Alexithymia and Aggressive Behaviors

The frustration–aggression model [7,9] and the social learning theory [12,13] mentioned above allow an initial conceptual differentiation of RA and PA and of the differential role of emotion regulation for the two. In this regard, the suggestion posed by Farah et al. [31] seems of particular relevance, as they suggest that while the understanding and identification of one’s emotions is important for behavioral inhibition and the regulation of impulsive aggression associated with RA [32], it does not seem the case in the context of predatory, pre-meditated aggression. Accordingly, a specific emotion regulation deficit, namely alexithymia, should be regarded. Alexithymia is defined as a personality trait associated with an impairment in the processing, regulation, and communication of emotions [33,34,35]. It represents a specific emotion regulation difficulty that is well associated with specific neurological deficits in the prefrontal cortex and the amygdala in particular [36]. Individuals with heightened alexithymia traits show specific difficulties in identifying, describing, and experiencing subjective feelings and emotions, with a stimulus-bound, externally orientated, cognitive style (i.e., externally oriented thinking) [37,38]. Because of these specific difficulties in emotional processing, individuals showing alexithymia traits might experience increased unspecified negative emotions and effects [39,40]. Accordingly, specifically during adolescence, greater alexithymia was shown to associate with adjustment problems, referred to externalizing problems characterized by aggressiveness, opposite-deviant, and delinquent behaviors [25,41,42,43,44]. Furthermore, it is also associated with heightened hostility and anger [45] as well as increased aggressiveness and aggressive behaviors [25,30,41,42,43,46]. In this regard, it is worth noting it is also associated with heightened impulsivity [25], which is particularly relevant considering the conceptual distinction between RA and PA and their different correlates. Indeed, the physiological arousal that might result from the negative and unspecified emotionality associated with alexithymia might more likely and intensively lead to reactive, impulsive, and aggressive behaviors [11,31,47], rather than favoring pre-meditated, goal-directed aggressive acts. However, research on alexithymia and aggressive behaviors has mainly focused on impulse-related aggressiveness [25] and RA [31] instead of on pre-meditated, antisocial-like aggression such as PA.

### 1.3. Parental Attachment and Its Association with Alexithymia and Aggressive Behaviors

In order to further the understanding of the similarities and differences between RA and PA, the role played by adolescents’ attachment should be taken into account, particularly considering its role in the development of adaptive regulation capacities. Attachment refers to a deep and lasting emotional and affectional bond established through dyadic interactions with the attachment figures during infancy [48,49], which guides the development of youth’s internal working models (IWM). These IWM subsume the representation of the self and others, as well as of relationships and of ways of interacting [48,50,51,52]. As such, a secure attachment, characterized by more adaptive IWM, is considered pivotal to support the development of flexible and adaptive self- and co-regulation capacities. Accordingly, it was shown that attachment does shape emotion regulation capacities, as reflected in individual differences in its neural, behavioral, and cognitive correlates [53,54]. Referring specifically to the neural circuits influenced by attachment, they regard the interaction between higher and lower order structures, specifically the medial prefrontal cortex and the amygdala, respectively [55]. The adaptive maturation of these circuits is important to support the child’s willful control of the cognitive–affective mechanisms useful to emotion regulation [53]. These structures are indeed akin to those shown to be deficient in individuals presenting alexithymia traits [36], which highlights the influence of parental attachment upon alexithymia. Accordingly, research studies evidenced that alexithymia significantly mediated the association between insecure attachment and adolescents’ psychosocial symptoms [56,57,58] as well as emotion (dys)regulation more broadly [59]. In this regard, it should be noted that, although the adolescent struggles for individuation and autonomy from the parental figures during this specific developmental period, the parent–adolescent attachment relationship remains significant and predictive of adolescents’ mental health outcomes [60] and aggressive behaviors [61,62,63,64,65,66]. In this regard, secure attachment has emerged as a significant moderator of adolescents’ anger expression, thus associated with a reduction in the occurrence of aggressive behaviors [62,63,64,65,66]. On the other hand, insecure attachment correlates with higher levels of aggression [67], which is not surprising considering the above-mentioned close link between parental attachment and youth’s capacity to self-regulate.

Differentiating specifically between attachment to mothers vs. father, no sound conclusions on adolescents’ preferred attachment figure can though be yet drawn [68,69,70,71,72,73]. Indeed, although mothers are still somewhat considered as the primary parental figure in child rearing [69,72], fathers play a significant role in youth adjustment [74,75] and adolescent development (for a review, see [76]). As such, differentiating between these two figures might differently account for adolescents’ emotion regulation capacities and difficulties and aggressive behaviors [63,77]. Meta-analytic evidence [78] supported the significant influence of the parenting behaviors of both mothers and fathers upon adolescents’ aggressive behaviors. Nonetheless, it is worth noting that various studies [79,80,81,82,83] investigated the association between attachment to parents and youth aggression at large, while fewer have specifically investigated the differential influence of attachment to maternal vs. fathers on adolescents’ aggression. These studies suggested that insecure attachment to fathers, and paternal practices in general, greatly predict increased aggression during adolescence [4,84,85]. Yet, the protective role of secure attachment to mothers was also reported regarding adolescents’ aggressiveness [63,86], which was also supportive of adolescents’ prosocial behaviors [86].

Even fewer are the findings referred to the association between adolescents’ perceived attachment to mothers vs. fathers and RA and PA specifically [63,87]. Nonetheless, findings showed that reduced secure attachment to mothers is significantly associated with adolescents PA as well as RA, while insecure paternal attachment only with PA [63]. Others have instead highlighted the greater influence of maternal variables upon PA compared to RA, [88] highlighting that maternal criticism was bidirectionally associated with PA only. In this regard, it should be noted that the available literature has greatly investigated the role played by maternal and paternal behaviors and attitudes, for instance, referred to their parenting style [77,89,90,91], parental control, and monitoring [88,92] compared to attachment specifically. However, based on the aforementioned and mindful of the biopsychosocial peculiarities of the adolescence period, the investigation and understanding of the influence of adolescents’ perceived attachment to their mothers and fathers might allow the provision of information useful to prevention interventions. This would ultimately support the development of more effective treatment packages that can more broadly address adolescents’ emotional and behavioral regulations as well as the functions played by RA vs. PA.

### 1.4. Aims and Hypothesis

Based on the reviewed literature, the current study intends to further the understanding of the interplay between adolescents’ perceived attachment to parents, emotion regulation difficulties referred to as alexithymia, and RA and PA. RA vs. PA is shown to play different functions [5,6,7], whereby RA would be more emotion-driven, while PA cooler and pre-meditated as highlighted by their previously-reported different correlates. However, since no study has to current knowledge jointly and empirically evaluated their interplay in adolescence, the present study aimed to exploratorily investigate the differential influence of maternal vs. paternal attachment upon RA vs. PA. Furthermore, the mediating role of adolescents’ alexithymia levels will also be investigated since, as outlined throughout the previous paragraphs, attachment to parents was shown to significantly influence youth’s regulation capacities [53,54,56,57,58] with repercussions on behavior. However, there is no consensus on the differential role played by maternal vs. paternal attachment upon emotion regulation difficulties and particularly on the more specific alexithymia and, as such, this will be exploratorily investigated. Overall, as an applied consequence of the current study’s results, the intent is to provide evidence useful to clinical practice as well as to deepen understanding of the differential functions and mechanisms contributing to RA vs. PA in adolescence.

In light of the above, it was expected that, albeit with their differential function, (a) RA and PA would be significantly associated [6,14,15]. Moreover, mindful of the literature inconsistencies, yet given the historical role played by mothers in child-rearing as well as the evidence reporting that mothers show a warmer and more comprehensive parenting style [93], it is expected that (b) mothers, compared to fathers, would result as adolescents’ preferred parental figure [68,69,72]. Coherent with the previously outlined frustration–aggression model [7,9] (c), it was then expected that alexithymia would result as overall more influential for RA compared to PA [31]. However, given the reported literature inconsistencies, there was no further expectation regarding the direct and indirect effects—the latter mediated by alexithymia—of maternal vs. paternal attachment upon RA vs. PA. For the same reason, gender differences among adolescents in all considered variables were exploratorily assessed, and adolescents’ gender was solely included as a covariate within the mediational models.

## 2. Materials and Methods

### 2.1. Participants and Procedure

Participants were 453 Italian adolescents aged 15–19 years (Mage = 16.48, SD = 0.69; 33.6% males). This study was conducted in 2017, and the sample included students recruited from high schools (grades 10th to 14th) located in northern Italy and belonging to middle-class families and living in either urban or suburban districts (SES, [94]). More than 90% of the participants came from two-parent households. Only a few participants (<5%) reported receiving previous psychological support for mild problems (e.g., academic problems, short-term emotional disturbance), while none was ever hospitalized or took medicines because of psychiatry symptoms in the previous two years.

Participants completed paper-and-pencil self-report measures during regular school hours. Questionnaires were administered by a trained psychology master student under the supervision of a certified psychologist, and participants were instructed to answer each question, thereby avoiding omissions; only complete questionnaires were considered by the current study. Before participation in the study, signed informed consent by the minors’ parents or by the participant themselves, if already over 18 years of age, were required. Before participation, participants provided oral consent. Participants were informed that participation was voluntary and that data were collected anonymously; they were also informed that they could withdraw from the study at any moment without repercussions ensuring the non-utilization of their data. The study was conducted in compliance with the Declaration of Helsinki (Italian law 196/2003) and was approved by the University Ethical Committee (1523/15).

### 2.2. Measures

#### 2.2.1. Inventory of Parent and Peer Attachment-Revised-IPPA-R

The IPPA-R [95,96] assesses adolescents’ perceived attachment to their parents, separately for mothers and fathers, and peers based on a 5-point Likert scale (from “0 = never” to “4 = always”). IPPA-R includes 25 items measuring the extent of Trust, Communication, and Alienation separately for each of the attachment figures with parallel wordings of items, thereby providing a measure of attachment for each as well as an IPPA-R total score; for the current study, only attachment to mothers and fathers were considered. High scores indicate a stronger and more secure attachment. Sample items are “I am angry at my mother” (reversed score) and “my mother respects my feelings”. This instrument has been extensively used in the literature; the Italian validation of the tool showed overall adequate psychometric characteristics and was employed in the present study [97]. The internal consistency assessed on the current sample was α = 0.94 (95%, ICC 0.94–0.95) for IPPA-R father and α = 0.94 (95%, ICC 0.94–0.95) for IPPA-R mother.

#### 2.2.2. Toronto Alexithymia Scale 20-TAS-20

The TAS-20 ([98,99]; Italian validation [100]) is a self-report measure consisting of 20 items rated on a 5-point Likert scale. TAS-20 total score was considered, with scores that can range between 20 and 100; higher scores denote greater alexithymia. Examples of items are “I am often confused about what emotion I am feeling”, “I prefer just to let things happen rather than to understand why they turned out that way”, “It is difficult for me to find the right words for my feelings”. The TAS-20 shows adequate validity and reliability (α = 0.81; r = 0.77). The Italian version also demonstrates good internal consistency (Cronbach’s alpha of 0.75 and 0.82 in normal and clinical groups, respectively). In the current study the internal consistency was α = 0.82 (95%, ICC 0.79–0.84).

#### 2.2.3. Reactive–Proactive Aggression Questionnaire—RPQ

The RPQ [6] is a 23-item questionnaire designed to measure RA and PA in children and adolescents; 11 items measure RA while 12 items measure PA. Items are rated on a 3-point Likert scale (0 = never, 1 = sometimes, 2 = often); items do not refer to a specific period but simply ask how often respondents have engaged in a particular behavior. Items are summed to yield a RA final score and a PA final score. Previous research using the RPQ has reported high internal consistency, with α ranging from 0.89 to 0.91 [6]. The RPQ was validated in Italy by Fossati et al. [30], of use in the current study. Example items are “how often do you react with anger when provoked by others” (item 3) for RA and “how often do you use violence to force others to do what you want” (item 12) for PA. Internal consistency in the present study was α = 0.77 (95%, ICC 0.74–0.80) for RA and α = 0.70 (95%, ICC 0.64–0.74).

### 2.3. Data Analyses

Statistical analyses were performed using SPSS (IBM Corp, Armok, New York, NY, USA) and R (R_Core_Team, Vienna, Austria). The Shapiro–Wilk test was performed highlighting that the sample does not present a normal distribution. Accordingly, the Mann–Whitney U non-parametric test for independent samples was used to assess gender differences among adolescents. The Wilcoxon non-parametric test for paired samples was instead used to assess differences in adolescents’ perceived attachment to mothers vs. fathers. Correlation analyses were carried out using Spearman rho to assess the associative pattern among IPPA-R mother and father, RA, PA, and TAS-20; correlations were interpreted only when effect size > 0.20 [101].

Through SPSS macro (PROCESS; [102]), four mediation models (PROCESS Model 4) were performed, assessing the direct and indirect effects of attachment to mothers (IPPA-R Mother) vs. fathers (IPPA-R Father) on either RA or PA considering the mediating role of alexithymia (TAS-20); adolescents’ gender was included as a covariate. The bootstrapping method [102,103] was applied, and 5000 bootstrap samples were drowned from the full data. The bootstrapping method is a high-power non-parametric test for hypothesis testing that poses no assumptions on the sample distribution. It generates a series of empirically derived samples, starting from a sample of n size during which observations are resample with replacement and the model effects are re-calculated throughout these resampling processes, which were repeated here 5000 times. Through this method a bootstrap confidence interval that respects the sample irregularities is constructed, providing more accurate information [102]. In the current study, a 95% confidence interval was used to determine the significance of the mediating effect. A significant effect would be identified if the confidence interval excluded 0. Moreover, it should be noticed that, notwithstanding the cross-sectional nature of the current study, the terms “effect”, “influence”, or “mediation” are used, yet not to imply a causal relationship among variables. More specifically, as can be found in other cross-sectional studies performing mediation analysis [25] and following Hayes’ position [102], the mentioned terms were used in accordance with the Ordinary Least Squared (OLS) regression method adopted [102]. Notably, in multivariate regressions, the association of an independent/criterion variable on the dependent one is assessed while controlling for the effect of all others, thereby controlling for the shared variance among the independent variables included within the models. As such, these terms were used to assess the unique contribution of each independent variable upon the dependent one while not implying the presence of causal relationships among the considered variables.

## 3. Results

### 3.1. Preliminary Analysis

Means, standard deviation, and Mann–Whitney *U* test results of all considered variables are reported in Table 1. Values refer to the overall sample, as well as males and females separately. Moreover, the median and scores range of all variables referred to each quartile of their distribution are shown in Table 2.

Significant gender differences among adolescents have emerged for PA, attachment to mothers, and attachment to fathers. Specifically, males showed significantly higher PA and attachment to fathers compared to females, while females present significantly greater attachment to mothers compared to males. Paired sample Wilcoxon test comparing attachment to mothers vs. fathers as assessed on the overall sample also showed significant differences. Notably, the mother resulted as the attachment figure toward which adolescents altogether perceived greater attachment security (attachment to mothers vs. father stat = 74,714; df = 451; *p* < 0.001).

Correlations are shown in Table 3. RA and PA significantly and positively correlated, showing a large effect size. Moreover, RA was positively and significantly correlated with alexithymia and negatively and significantly correlated with adolescents’ attachment to mothers and fathers. Alexithymia as well was significantly and negatively correlated with attachment to both mothers and fathers, albeit the letter presents a small effect size (<0.20). All variables’ correlations with RA showed at least a medium effect size.

Regarding PA, it was significantly and negatively correlated with alexithymia, although showing a small effect size. PA was also significantly and negatively correlated with attachment to mothers with medium effect size, while to fathers with a small effect size (<0.20).

### 3.2. Mediational Models

#### 3.2.1. The Mediating Role of Alexithymia in the Association between Attachment to Mothers and RA vs. PA

**Figure 1:**
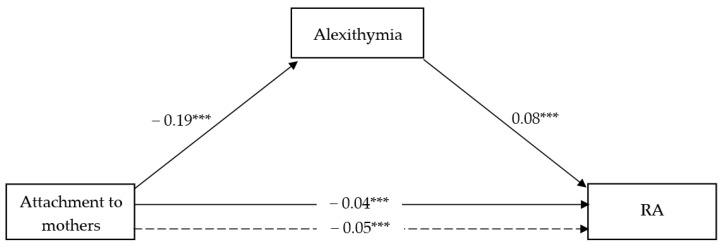
Attachment to mothers and RA. *** *p* < 0.000; dotted line = Total effect.

**Figure 2:**
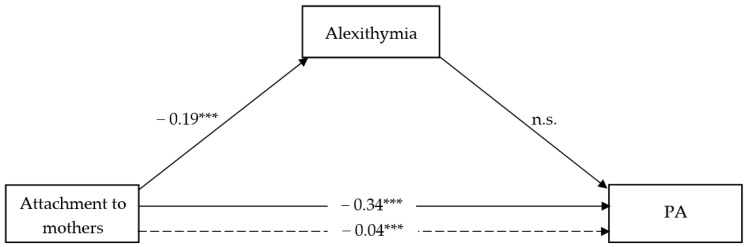
Attachment to mothers and PA. *** *p* < 0.000; ns = not significant; dotted line = Total effect.

The full models shown in Figure 1 and Figure 2 accounted for 7.24% and 10.79% of the total variance in RA vs. PA scores, respectively. In particular, in both models, attachment to mother showed a negative and significant effect on alexithymia (β = −0.19; t = −7.08; CI = −0.25, −0.14), with adolescents’ gender as covariate resulting significant (β = 2.50; t = 2.55; CI = 0.58, 4.42). Moreover, attachment to mother showed a negative and significant direct effect on RA (β = −0.04; t = −4.06; CI = −0.06, −0.19) as well PA (β = −0.03; t = −5.12; CI = −0.05, −0.02), and alexithymia also showed a significant, albeit positive, effect on RA (β = 0.08; t = 5.44; CI = 0.05, 0.11), yet not on PA. As such, a significant indirect effect of attachment to mother on RA through lower alexithymia has emerged (β = −0.02; CI = −0.02, −0.01) while no indirect effect was shown regarding PA. Adolescents’ gender included as a covariate was not significant in either model. 

#### 3.2.2. The Mediating Role of Alexithymia in the Association between Attachment to Fathers and RA vs. PA

**Figure 3:**
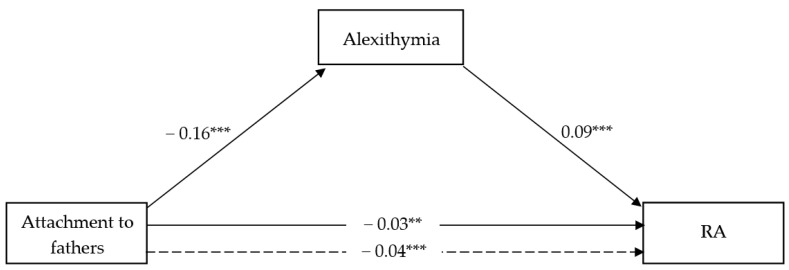
Attachment to father and RA. ** *p* < 0.01; *** *p* < 0.000; dotted line = Total effect.

**Figure 4:**
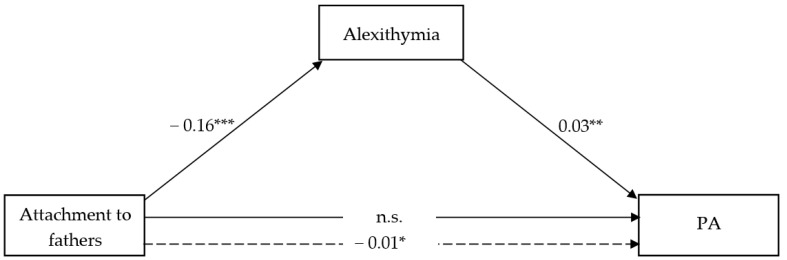
Attachment to father and PA. * *p* < 0.05; ** *p* < 0.01; *** *p* < 0.000; ns = not significant; dotted line = Total effect.

The full models shown in Figure 3 and Figure 4 accounted for 5.08% and 9.01% of the total variance in RA vs. PA scores, respectively.

Similarly to what observed for attachment to mother, attachment to fathers showed a significant negative effect on alexithymia (β = −0.16; t = −6.36; CI = −0.21, −0.11) in both models, and a negative and significant direct effect on RA (β = −2.03; t = −3.18; CI = −0.04, −0.01). However, no direct effect of attachment to fathers on PA has emerged. Nonetheless, alexithymia showed a significant and positive effect on both RA (β = 0.09; t = 5.83; CI = 0.06, 0.12) and PA (β = 0.03; t = 2.61; CI = 0.01, 0.05), thereby resulting in a negative and significant indirect effect of attachment to fathers on RA (β = −0.01; CI = −0.02, −0.01) as well as PA (β = −0.01; CI = −0.009, −0.01) through lower alexithymia. Adolescents’ gender as a covariate was never significant in the RA model, while it was significant in the PA’s full model.

## 4. Discussion

The current study was focused on exploratorily investigating the interplay between adolescents’ attachment to parents—separately accounting for adolescents’ attachment to mothers vs. fathers—and alexithymia upon RA and PA during adolescence.

Empirically, during adolescence, RA and PA were revealed to be highly associated [5,6,15], which was indeed supported by the present findings. Nonetheless, the current study has highlighted significant differences, whereby male adolescents showed significantly greater PA compared to females, while no gender difference has emerged for RA among adolescents. This finding is in line with PA correlates, which were all shown to be greater and more prevalent among males compared to females and regard antisocial personality traits [104,105,106], conduct disorders [106,107,108], and psychopathy traits [109]. Accordingly, most of the literature on aggressive behaviors, psychopathy, and criminality relied on male samples [17,26,110,111] On the other hand, referring to RA, the comparable levels of RA reported by male and female adolescents might be given by adolescents’ overall poor regulation capacities [3], which are indeed associated with RA, as also highlighted by the current findings. Specifically, alexithymia was shown to be overall more influential upon RA compared to PA. This is coherent with past evidence highlighting that adolescents’ difficulties in emotional processing and regulation, such as those referred to as alexithymia, are associated with increased aggressive behaviors [25,27,43,85] and with RA in particular [3,31]. In this regard, it is worth noting that past findings highlighted that alexithymia and RA, yet not PA, present shared neurobiological determinants, whereby their association would result from hyperactivation of the right hemisphere while being fully mediated by the right amygdala volume [31]. Higher alexithymia is associated with more intense emotional experiences of negative emotions and affects [112,113] and greater right hemisphere activation, which represents a biological vulnerability that guides attention toward negative stimuli [31,114,115]; as such, and in accordance with Farah and colleagues [31], the increased and misregulated negative emotions associated with alexithymia might then lead to greater impulsive aggressive behaviors.

Mediation analysis has further evidenced the protective role of adolescents’ secure attachment to parents, highlighting differences in the contribution posed by attachment to mothers vs. fathers upon RA vs. PA as mediated by alexithymia. In particular, findings showed that adolescents’ perceived attachment security toward both parents has a protective role toward the impulse-driven RA, which is then favored by reduced alexithymia. Secure attachment to parents indeed supports emotion regulation capacities as influencing the development of the specific neural circuits (i.e., medial prefrontal cortex and the amygdala) responsible for emotion regulation, and that has indeed shown to be deficient both in the context of alexithymia [36] and RA [31]. These results also support past findings [11,31,116,117], highlighting that understanding and identifying ones’ emotions is important for behavioral inhibition and to regulate impulsive aggressive reactions while not greatly contributing to the more willful and pre-meditated PA [11,31]. These findings are particularly relevant for adolescents since their typical and specific development foresees still scarce regulation emotional capacities, which are in turn associated with heightened physiological arousal rendering adolescents more vulnerable to RA overall [3]. This should warrant caution since both RA and emotion regulation difficulties referred to as alexithymia are associated with increased internalizing problems subsuming anxiety and depression symptoms [41,42,110,118,119]. This might thus further challenge adolescents’ mental health and mental health outcomes later in adulthood. Accordingly, and mindful of the shared neural correlates between RA, alexithymia, and the regulation capacities resulting from attachment security [31,36,55], clinical practice should try and make the most of adolescents’ neural plasticity [3]. These findings highlighted the need and stressed the usefulness of implementing prevention practices for RA by fostering adolescents’ capacity to better comprehend, verbalize, and regulate emotions to reduce alexithymia. Moreover, prevention intervention might also be directed to parents to favor a secure attachment that can support youth regulation capacities and the willful use of these competencies [53,120].

A more complicated picture has instead emerged regarding the interplay between adolescents’ attachment and alexithymia upon PA, which showed some differences in the contribution posed by attachment to mothers vs. fathers. Specifically, a more secure attachment to mothers has shown a protective role toward PA, which was though not supported by adolescents’ capacities to recognize and process emotions. On the other hand, a more secure attachment to fathers emerged as protective toward increased PA only by influencing adolescents’ alexithymia levels. These findings showed that the positive bond perceived toward the maternal figure could mitigate PA behavior in adolescence, regardless of adolescents’ emotional awareness. However, results also seem to suggest that affects and emotional awareness, thus characterized by a more internally oriented thought modality, might instead be protective toward PA when favored by secure attachment to fathers. Therefore, these results seem to suggest that in the context of pre-meditated, cool aggressive behaviors such as PA, the bond perceived with fathers and related attachment guides adolescents’ affects and emotions’ perception and awareness, while the quality of the bond with mothers seems to influence adolescents’ affects greatly. This hypothesis is in line with the reported difference in parental practices and parenting style shown by mothers compared to fathers. Notably, a parenting style and attitude characterized by warmth, comprehension, and affectivity were shown to foster adjustment among aggressive adolescents [90], which were in turn associated with better adolescent–parent relationship quality [91,121]. This sort of parenting style, thus more authoritative, seems more prevalent among mothers as opposed to fathers; the latter is reported as more authoritarian and thus harsher and more “rule-imposing” [93]. Accordingly, adolescents, in the current study, reported being greatly attached to their mothers compared to their fathers, thereby perceiving with the maternal figure a greater bond and more positive relationship quality characterized by greater communication and trust [97]. Notwithstanding, it is worth noting that the current findings also pointed to gender differences among adolescents in their attachment to parents. Specifically, during adolescence, females showed significantly greater attachment to mothers, while males to fathers. However, although these tentative results showed statistically significant differences, male and female scores of their attachment to either mother or fathers are close, suggesting a limited clinical valence of these differences. However, future studies are needed to further the understanding of gender differences in adolescents’ attachment to parents.

Altogether, the current findings point to the important buffering role of emotional awareness (i.e., low alexithymia traits) as favored by a secure attachment to parents upon adolescents’ RA. On the other hand, PA was differently influenced by adolescents’ attachment to parents and subsequently alexithymia. Although still preliminary, these findings add knowledge relevant to research investigating the association between emotional processes (i.e., alexithymia) and PA, which are still empirically very scarce compared to studies focused on RA [25,31]. Nonetheless, the current findings provide evidence useful for clinical practice and to the development of preventive programs and interventions during adolescence. Mindful that the current study focused on community adolescents with no psychiatry history, it is anyways worth noting that national guidelines [122] referred to evidence-based treatments for adolescents showing clinically relevant aggressive behaviors (e.g., conduct disorders, oppositional defiant disorder) and antisocial behaviors in general, all stressed the importance of including parents within these interventions. Indeed, providing adequate treatment during youth while guiding parents regarding their parenting style and behaviors is expected to reduce the likelihood of showing more severe aggressive behaviors in adulthood, thereby also reducing the resulting social cost [123]. In this regard, new and tailored preventive research intervention projects, such as the LOOK@ME project in north Italy [124], are being promulged to support adolescents’ regulation capacities and the adoption of better regulation strategies, thus supporting their well-being both during adolescence and later in adulthood while furthering empirical investigations [125]. Furthermore, these sorts of projects, promulgated within the school context, have the added value of favoring a support network that includes both parents and teachers, indeed coherent with suggestions from national guidelines and referred to the prevention and treatment of aggression-related behaviors and disorders [122].

### Limitations and Future Research

This study has some limitations that should be acknowledged. First of all, it was carried out using solely self-report measures, particularly critical regarding the assessment of aggressive behaviors [14]. Moreover, only adolescents’ perceived attachment to parents was considered; thus, other parental measures need to be adopted to extend these exploratory and preliminary findings. It should also be noted that difficulties in emotion regulation were inferred from adolescents’ alexithymia level, which represents a specific construct internal to the broader and multidimensional emotion regulation construct [126]. Accordingly, future studies should include other measures of emotion regulation, thereby more specifically accounting for emotion (dys)regulation facets [126]. Future research should also extend the current evidence by specifically investigating gender differences in adolescence, particularly since some preliminary evidence has emerged regarding adolescents’ gender differences in both aggression and attachment to parents. Moreover, gender included as a covariate within the mediational models was significant, which suggests the significant contribution of gender upon the performed models. Notwithstanding these limitations, a strength of the current study regards its sample size, which supports results reliability. Finding’s reliability is further favored by the bootstrapping method used for hypothesis testing within the mediational models performed. Indeed, as previously reported, this method shows high power, and thereby provides more accurate and reliable results. In this regard, it should be noted that a further limit of the current study is its cross-sectional design which limits findings informativeness regarding causal links between the considered variables. As such, the terms “influence”, “effect”, “mediates”, and “protective” were employed in line with the mediational models performed, which allow the assessment of the unique contribution of each independent variable upon the dependent one, thus also informing on the direction of such effect; nonetheless, these terms were not used to implying causal relationships between the considered variables. Accordingly, future studies should try and replicate the emerged evidence using a longitudinal design, which would on the other hand allow inferences about causality. Longitudinal studies are indeed crucial to enhance knowledge about the unfolding, over time, of the link between familial precursors and the long-term consequences associated with aggression sub-types and functions.

## 5. Conclusions

This study provides compelling evidence that the distinction between RA and PA is useful and deserving of further research attention, particularly regarding their association with adolescents’ attachment to mothers vs. fathers and emotional processes. Much research remains to be conducted to understand further similarities and differences in RA and PA during adolescence; more time and labor-intensive observational, as well as laboratory-based investigations, and more longitudinal studies, are required. Notwithstanding, the findings from the current study highlighted how RA and PA could be understood further by considering both adolescents’ inability to identify and understand emotions as well as their specific and differential attachment to their parents.

## Figures and Tables

**Table 1:** Variables’ mean and standard deviation, and Mann–Whitney *U* test results.

	Overall		Males		Females			
	M	SD	M	SD	M	SD	Stat (451)	*p*
Reactive Aggression	6.43	3.29	6.42	3.30	6.44	3.29	22,750	0.99
Proactive Aggression	1.95	2.41	2.76	2.70	1.54	2.15	29,708	<0.00 ***
Attachment to father	87.68	18.88	90.55	15.98	86.23	20.06	25,279	0.05 *
Attachment to mothers	97.33	16.83	95.43	15.17	98.28	17.56	19,376	0.01 **
Alexithymia	51.36	9.15	50.11	9.15	52.00	10.33	20,213	0.06

Notes. * *p <* 0.05; ** *p <* 0.01; *** *p* < 0.00.

**Table 2:** Variables’ median and values range within each quartile.

	Q1 Md (R)	IQR Md (R)	Q3 Md (R)
Reactive Aggression	4 (0–6)	6 (7–8)	11 (9–18)
Proactive Aggression	0 (0–0)	1 (1–2)	4 (3–18)
Attachment to father	63 (36–75)	91 (76–100)	108 (101–125)
Attachment to mothers	79 (41–88)	99 (89–108)	116 (109–125)
Alexithymia	40 (20–44)	51 (45–58)	64 (59–86)

Note. Md = median; R = Range; IQR = Q1–Q3.

**Table 3:** Spearman’s rho Correlations.

	1. RA	2. PA	3. Paternal Attachment	4. Maternal Attachment	5. Alexithymia
2.	0.53 **	-			
3.	−0.24 **	−0.16 **	-		
4.	−0.28 **	−0.31 **	0.47 **	-	
5.	0.31 **	0.17 **	−0.29 **	−0.35 **	-

Notes. ** *p <* 0.01.

## Data Availability

The data presented in this study are available on request from the corresponding author.
